# A Bidirectional Barbed Suture Technique for Secure Entry-hole Closure in Robotic Overlap Esophagojejunostomy: Initial Experience

**DOI:** 10.31662/jmaj.2026-0137

**Published:** 2026-07-24

**Authors:** Hironori Tsujimoto, Keita Kouzu, Yoshihisa Yaguchi, Hiroyuki Horiguchi, Naoyuki Uehata, Risa Kariya, Asuma Ide, Seiichiro Fujishima, Takafumi Suzuki, Masahiro Takei, Hiroki Tashiro, Hideki Ueno

**Affiliations:** 1 Department of Surgery, National Defense Medical College, Tokorozawa, Japan

**Keywords:** esophagojejunostomy, entry hole, robotic surgery

## Abstract

**Background:**

Although minimally invasive total and proximal gastrectomy have been increasingly adopted for gastric cancer, anastomotic complications, particularly at the esophagojejunal anastomosis, remain more frequent than after distal gastrectomy. Anastomotic leakage or stenosis can delay postoperative recovery and the initiation of adjuvant chemotherapy, thereby potentially affecting oncologic outcomes. Reliable entry-hole closure during overlap esophagojejunostomy remains technically challenging, especially when luminal diameter mismatch exists between the esophagus and jejunum.

**Methods:**

We describe an entry-hole closure technique using a bidirectional barbed suture during robotic overlap esophagojejunostomy. Ten consecutive patients undergoing robotic gastrectomy for gastric cancer (five total gastrectomies with Roux-en-Y reconstruction and five proximal gastrectomies with double-tract reconstruction) were included. Following stapled overlap anastomosis, the entry hole was closed intracorporeally using a STRATAFIX^Ⓡ^ Spiral PDS Plus^Ⓡ^ bidirectional barbed suture. Suturing was initiated at the midpoint of the entry hole and proceeded continuously toward both the distal and proximal ends, allowing tension adjustment and full-thickness layer capture without the need for terminal knot fixation.

**Results:**

Entry-hole closure was successfully completed in all patients without conversion. The mean closure time was 15.6 ± 2.3 minutes. No patients experienced anastomotic leakage, stenosis, or other anastomosis-related complications during the postoperative course.

**Conclusions:**

This technique appears technically feasible and safe in this initial experience and may facilitate secure entry-hole closure during robotic overlap esophagojejunostomy.

## Introduction

Minimally invasive gastrectomy, including laparoscopic and robotic approaches, has become an established treatment for both early and advanced gastric cancer ^(1)^. Although 40.1% of all gastrectomies are open total and proximal gastrectomies, only 19.2% are performed laparoscopically. The number of minimally invasive total or proximal gastrectomies is increasing each year, and the percentage of postoperative complications in laparoscopic distal gastrectomy is 11.3%, whereas that for laparoscopic total gastrectomy is 19.3% ^(2)^. The incidence of postoperative complications in laparoscopic distal gastrectomy is comparable to that in open surgery; however, anastomotic complications are more common in laparoscopic gastrectomy for upper gastric cancer. Therefore, a safe and simple anastomotic procedure after minimally invasive total or proximal gastrectomies should be standardized.

We previously reported favorable outcomes for overlap anastomosis using a linear stapler after laparoscopic total and proximal gastrectomies ^(3)^. In this study, the entry hole of the stapler was closed using an intracorporeal interrupted hand-sewn technique with absorbable monofilament sutures. Barbed sutures are widely used in laparoscopic and robotic surgeries, including esophagojejunostomy after total or proximal gastrectomy, owing to their unique design, which allows the suture to advance unidirectionally without loosening. However, when there is a difference between the size of the holes in the esophagus and jejunum, it is difficult to reliably close the entry hole using a running suture, even when barbed sutures are used. Previously, we reported that bidirectional barbed sutures offer a safe and feasible alternative option for temporary closure of the entry hole for delta-shaped anastomosis in minimally invasive distal gastrectomy ^(4)^.

In this report, we aimed to describe the technical details and initial clinical outcomes of a bidirectional barbed suture technique for entry-hole closure during robotic overlap esophagojejunostomy.

## Ethics Approval and Consent to Participate

All protocols were approved by the Institutional Review Board of the National Defense Medical College (Permission number: 5240). Written consent was obtained from the patients prior to the study.

## Materials and Surgical Technique

This was a retrospective observational study of 10 consecutive patients who underwent robotic gastrectomy at our institution. All procedures were performed by surgeons experienced in minimally invasive gastric surgery, each having performed more than 300 robotic or laparoscopic gastrectomies prior to this study.

After a gastrectomy was performed along with an appropriate lymphadenectomy based on tumor stage, the esophagus was transected using a linear stapler. Depending on the type of surgery, Roux-en-Y reconstruction was used for total gastrectomy, and double-tract reconstruction was used for proximal gastrectomy. A small opening was made on the right side of the esophagus, and all layers were sutured with an absorbable suture to prevent insertion of the linear stapler into the submucosal layer (3-0 Vicryl, Ethicon, Tokyo, Japan) ^(3)^. The jejunum was transected intracorporeally, 20 cm distal to the ligament of Treitz, using a linear stapler. An additional 10 cm of the distal jejunum was resected to minimize excessive tension at the anastomotic site during the esophagojejunostomy. A small enterotomy was performed 5 cm distal to the stapler line on the antimesenteric side of the jejunal limb. One fork of the 45-mm linear stapler was inserted through this opening toward the oral side of the lumen, and the jejunal limb was raised to establish an esophagojejunostomy in an antecolic fashion. The other fork of the linear stapler was carefully introduced into the esophageal opening under the guidance of a nasogastric tube ^(3)^. Once both forks were inserted into their respective lumens, the two limbs were aligned to construct a side-to-side esophagojejunal anastomosis. Finally, completion of the esophagojejunal anastomosis and intraluminal hemostasis were confirmed.

Subsequently, the entry hole was closed using a bidirectional barbed suture (STRATAFIX^Ⓡ^ Spiral PDS Plus^Ⓡ^ Bidirectional; Ethicon). First, the centers of all layers of the entry holes on the esophageal and jejunal sides were sutured and ligated to account for differences in size between the two lumens (Figure 1). Continuous suturing was then performed from the center toward the distal side to ensure the inclusion of all layers. The entry hole size was then adjusted by tugging the sutured tissue toward the center from the distal side. Next, continuous suturing was performed from the center to the proximal side, followed by the same tugging technique (Figure 2). After suturing all layers, the edges were folded back, and the serosal muscle layer was sutured toward the center (Figure 3). This maneuver was carefully adjusted to ensure that the final closure diameter of the entry hole matched the surgeon’s preference.

**Figure 1.**
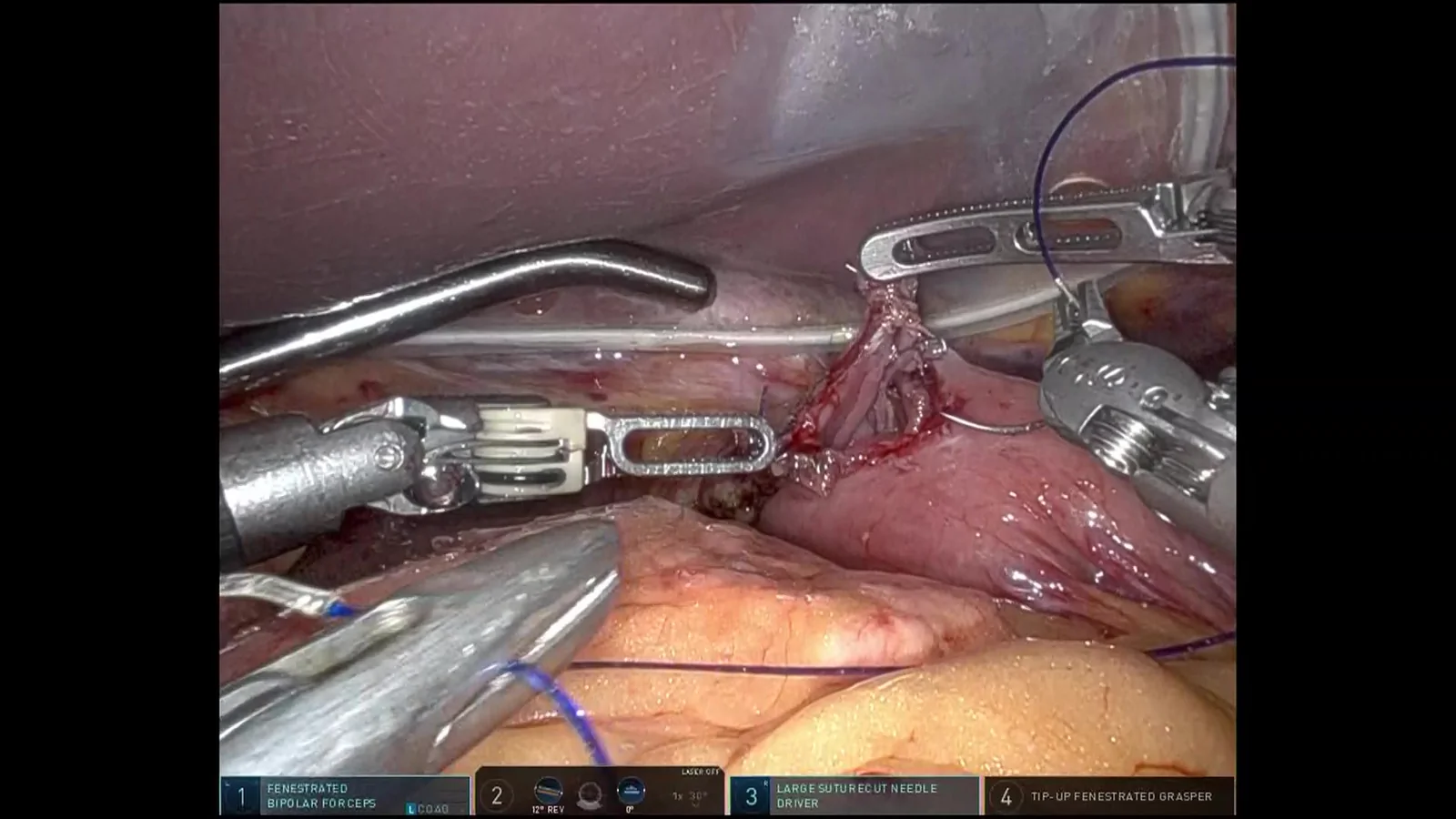
All layers at the center of the jejunum and esophageal walls are sutured with a bidirectional barbed suture (STRATAFIX^Ⓡ^ Spiral PDS Plus^Ⓡ^ Bidirectional, Ethicon, Tokyo, Japan).

**Figure 2.**
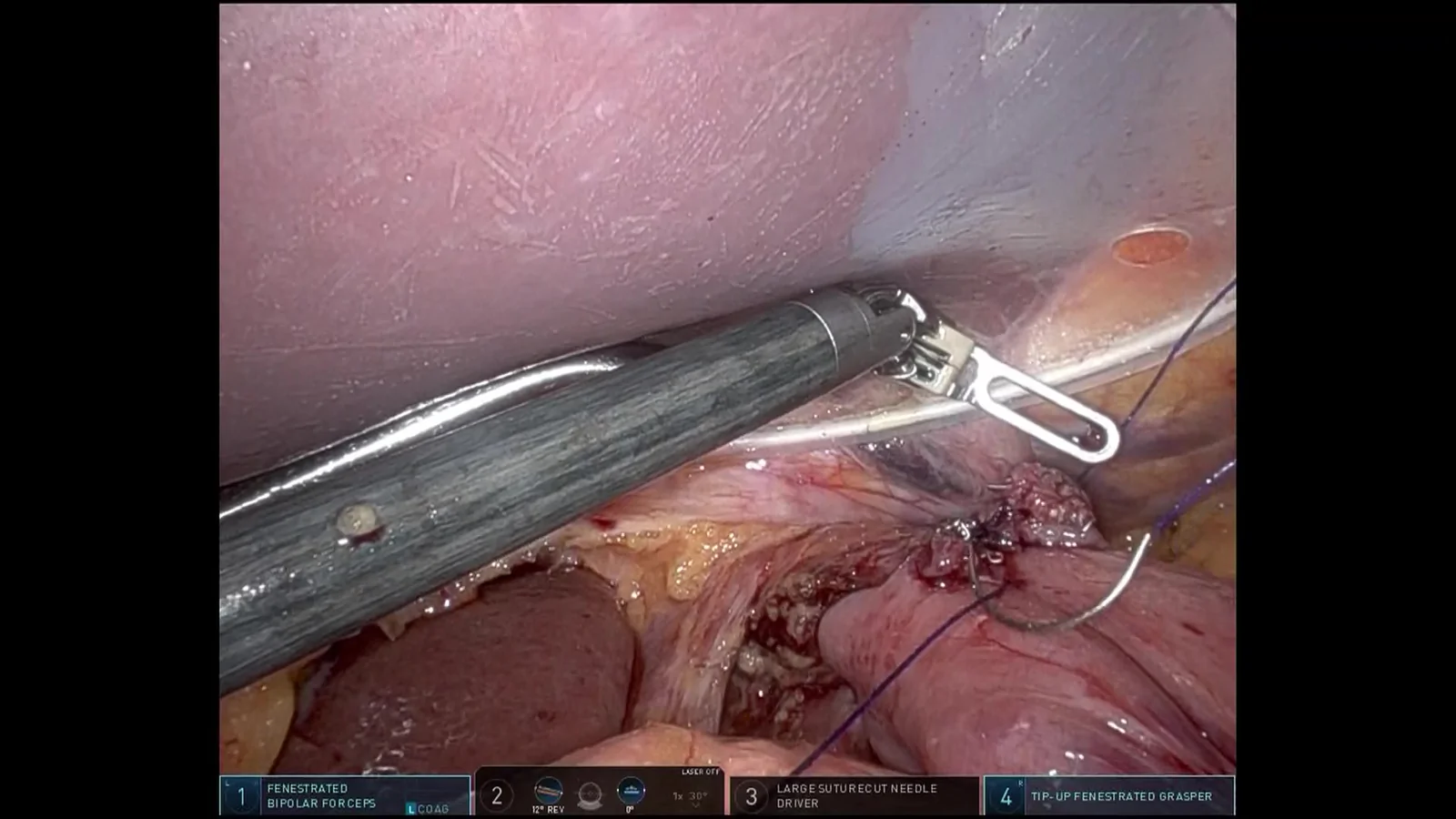
Continuous suturing is performed from the center toward the distal side, ensuring that all layers are included.

**Figure 3.**
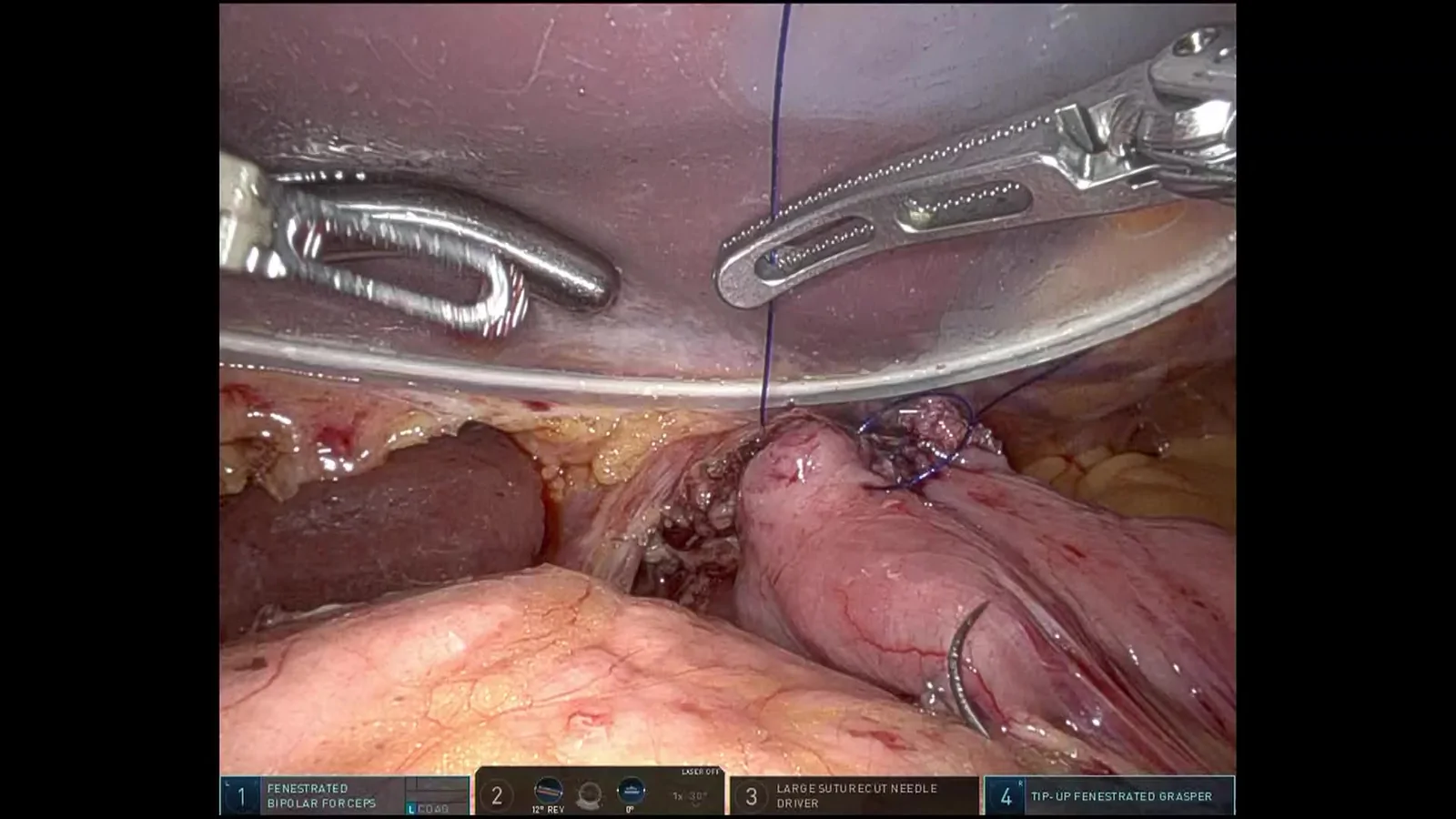
Next, continuous suturing is performed from the center toward the proximal side.

A leak test was conducted to ensure anastomotic integrity, and a Blake drain (19 Fr J-VAC drain; Covidien, Dublin, Ireland) was placed before completing the surgery.

The described technique was applied in 10 consecutive patients undergoing robotic gastrectomy for gastric cancer, including five total gastrectomies and five proximal gastrectomies. Entry-hole closure was successfully completed in all cases without the need for additional sutures or conversion to open surgery (Table 1). The mean time required for entry-hole closure was 15.6 ± 2.3 minutes. No anastomotic leakage, stenosis, or other anastomosis-related complications were observed during the postoperative period. All patients were discharged uneventfully. Anastomotic stenosis was assessed based on clinical symptoms, endoscopic findings, and the need for endoscopic dilation during follow-up (Figure 4).

**Table 1.** Surgical Outcomes of the Patients who Underwent Robotic Total or Proximal Gastrectomy.

Case	Age	Sex	BMI	cStage	Surgery	Operation time (minutes)	Closure time* (minute)	Days until first oral intake (day)	Postoperative hospital stays (days)	Postoperative complication
1	76	Male	22.4	III	TG	300	15	3	9	None
2	74	Male	16.6	II	TG	248	16	3	7	None
3	72	Male	22.0	I	PG	203	12	3	6	None
4	72	Male	20.2	III	TG	320	18	3	7	None
5	78	Female	20.1	III	PG	230	11	3	6	None
6	70	Male	26.9	III	PG	398	20	4	12	None
7	78	Male	27.7	IV**	TG	327	13	3	9	None
8	47	Male	26.3	IV**	PG	415	16	4	10	None
9	84	Male	23.7	I	PG	304	17	3	8	None
10	68	Female	18.7	IV**	TG	235	18	3	7	None
Mean ± SD	71.6 ± 6.0		22.5 ± 3.0			298.7 ± 54.4	15.6 ± 2.3	3.2 ± 0.3	8.5 ± 1.0	

*Closure time: time for entry hole closure for esophagojejunostomy. **Conversion surgery for Stage IV disease BMI: body mass index; PG: proximal gastrectomy; SD: standard deviation; TG: total gastrectomy.

**Figure 4.**
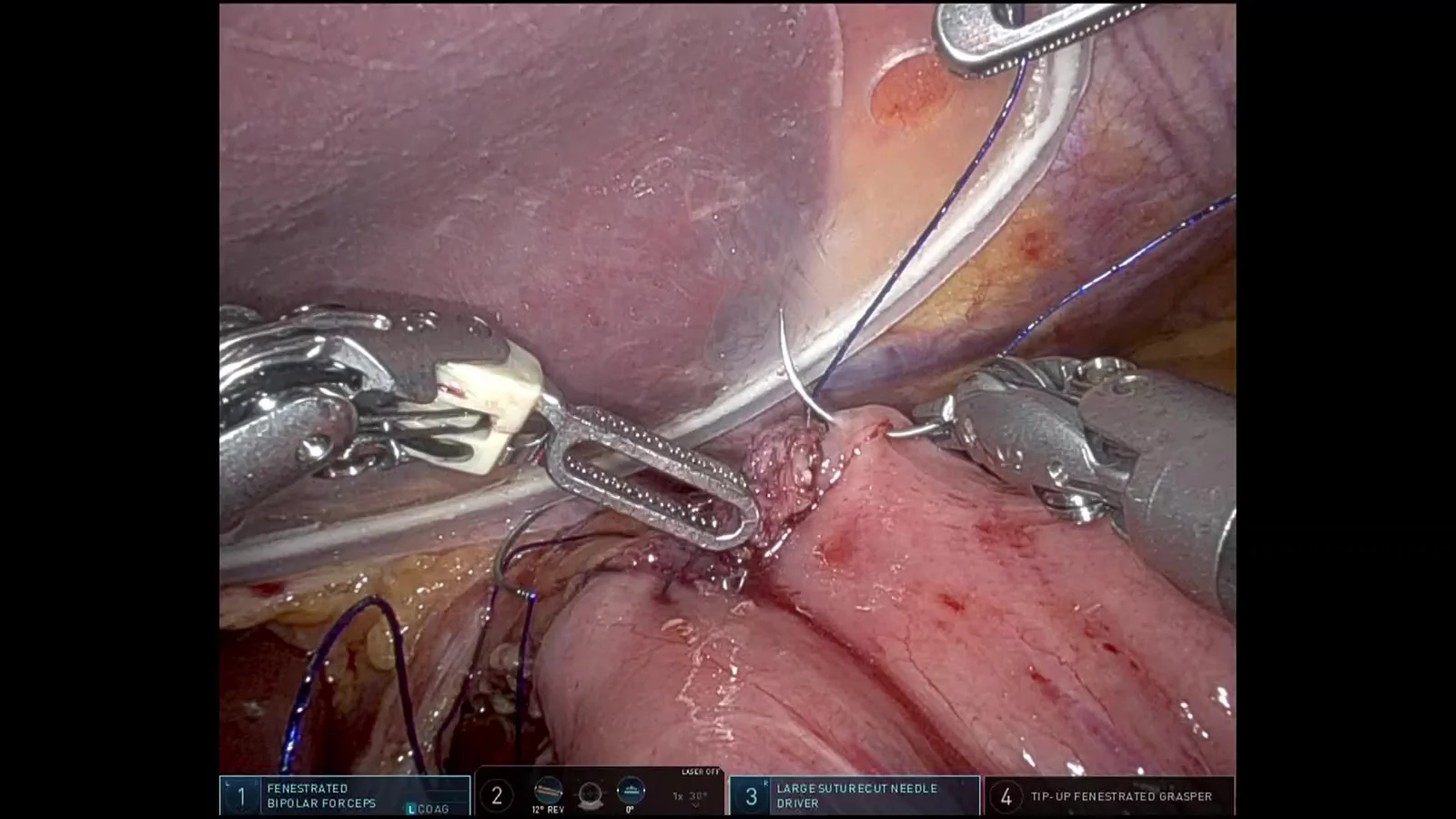
After all layers have been sutured, the ends are folded back, and the serosal muscle layers are sutured from distal to proximal to the center.

## Discussion

Anastomotic complications following esophagojejunostomy remain one of the most clinically significant challenges in minimally invasive total and proximal gastrectomy. Even minor leakage or stenosis can result in prolonged recovery, impaired nutritional status, and delayed initiation of adjuvant chemotherapy, all of which may compromise oncologic outcomes in patients with gastric cancer. In this report, we introduced the use of bidirectional barbed sutures to close the entry hole during overlap esophago-jejunal anastomosis. In our cases of overlap anastomosis, closure of the entry hole in the esophagojejunal anastomosis was completed without technical difficulty.

Although many surgeons have adopted barbed sutures for entry-hole closure in overlap anastomosis, we believe that bidirectional barbed sutures offer two potential advantages over conventional barbed sutures. First, both edges of the entry hole, where anastomotic leakage is more likely to occur, can be securely sutured. Many surgeons are aware that standard barbed suture has a loop at the end of the thread that cannot be secured. If this loop is positioned at the end of the entry hole, it may theoretically be associated with an increased risk of anastomotic leakage. In contrast, bidirectional barbed sutures allow secure suturing without such a loop at the ends of the entry hole. Second, bidirectional barbed sutures begin with suturing at the center of the entry hole. In esophagojejunal anastomosis, it is difficult to lift the jejunum in some cases. In such situations, the entry hole on the jejunal side tends to be larger and often requires special techniques to compensate for differences in caliber. By initiating the suture from the center, bidirectional barbed sutures provide a more adaptable approach. Moreover, surgeons often encounter situations in which anastomotic holes with large caliber differences require running sutures. In such cases, bidirectional barbed sutures may be useful not only for esophagojejunal anastomosis but also for other similar procedures.

This study has several limitations that should be acknowledged. First, the sample size was small, and the study was conducted at a single institution without a comparative control group. Therefore, the present analysis was not designed to evaluate superiority over existing techniques. The primary aim of this study was to establish the technical feasibility, safety, and procedural reproducibility of an entry-hole closure method using bidirectional barbed sutures during overlap esophagojejunostomy. Given the technical complexity of esophagojejunal reconstruction and the clinical impact of anastomotic complications, early demonstration of a consistent and reliable technique represents an essential first step before larger comparative or multicenter studies can be appropriately designed. In addition, only short-term outcomes were evaluated. The median follow-up period was 12 months. Further studies with larger cohorts and longer follow-up are required to determine whether this technique contributes to reduced anastomotic complications, such as anastomotic stenosis, and improved oncologic outcomes, including timely initiation of adjuvant therapy.

In conclusion, the present technique using bidirectional barbed sutures appears technically feasible and reproducible for entry-hole closure during robotic overlap esophagojejunostomy, particularly in situations involving luminal caliber mismatch. Further comparative studies with larger patient populations are warranted to validate its clinical benefits.

## Article Information

### Acknowledgments

We would like to thank Editage (www.editage.jp) for the English language editing.

### Author Contributions

Devised the surgical procedure: Hironori Tsujimoto. Conceived and designed this procedure: Hironori Tsujimoto, Keita Kouzu, Yoshihisa Yaguchi, Hiroyuki Horiguchi, Naoki Uehata, Risa Kariya, Asuma Ide, Seiichiro Fujishima, Takafumi Suzuki, Masahiro Takei, Hiroki Tashiro, and Hideki Ueno. Prepared the manuscript: Hironori Tsujimoto. Supervised the study: Hideki Ueno. All authors reviewed and agreed to the final submitted version of the manuscript.

### Conflicts of Interest

None

### Ethics Approval

All procedures involving human participants were in accordance with the ethical standards of the institutional and national research committees and the 1964 Helsinki Declaration and its later amendments. The study protocol was approved by the Institutional Review Board of the National Defense Medical College. (Permission number: 5240).

### Data Availability Statement

All data are available upon request from the corresponding author.

### Participant Consent

Informed consent for participation in the study or equivalents was obtained from all patients. The patient provided permission for the publication of this video. Patient anonymity has been maintained.
